# Effects of 24-h acute total sleep deprivation on physiological coupling in healthy young adults

**DOI:** 10.3389/fnins.2022.952329

**Published:** 2022-09-08

**Authors:** Hongyun Liu, Xiaohua Yu, Guojing Wang, Yi Han, Weidong Wang

**Affiliations:** ^1^Medical Innovation Research Division, Research Center for Biomedical Engineering, Chinese PLA General Hospital, Beijing, China; ^2^Key Laboratory of Biomedical Engineering and Translational Medicine, Ministry of Industry and Information Technology, Beijing, China

**Keywords:** sleep deprivation, autonomic nervous system, physiological coupling, heart rate variability, heartbeat-evoked potential

## Abstract

Sleep deprivation is associated with dysregulation of the autonomic nervous system, adverse cardiovascular events, cognitive and complex motor performance impairment. Less is known about the effects of acute total sleep deprivation (ATSD) on physiological coupling. We aimed to determine the effects of 24-h ATSD on the physiological coupling between complex subsystems by evaluating the cardiorespiratory, cardiovascular and cortico-cardiac interactions. This study enrolled 38 young healthy participants aged 23.2 ± 2.4 years. Multiple synchronous physiological signals including electrocardiography, photoplethysmography, bio-electrical impedance, electroencephalography, and continuous hemodynamic data, were performed over a baseline night after regular sleep and after a night with 24-h ATSD in the supine position. The magnitude squared coherence, phase synchronization index, and heartbeat evoked potential amplitudes, were obtained from 10-min synchronous physiological recordings to estimate the coupling strength between two time series. Parameters of hemodynamic characteristics and heart rate variability were also calculated to quantify autonomic regulation. Results indicated that the magnitude squared coherence (0.38 ± 0.17 vs. 0.29 ± 0.12, *p* = 0.015) between respiration and heart rate variability along with the magnitude squared coherence (0.36 ± 0.18 vs. 0.27 ± 0.13, *p* = 0.012) between respiration and pulse transit time were significantly decreased after 24-h ATSD. There were no significant differences (all *p* > 0.05) in phase synchronization indices, heartbeat evoked potential amplitudes as well as other analyzed measurements between baseline and 24-h ATSD states. We conclude that exposure to 24-h ATSD appears to weaken the cardiorespiratory and respiratory-cardiovascular coupling strength of young healthy adults. These findings suggest that physiological coupling analysis may serve as a complementary approach for characterizing and understanding the complex effects of sleep deprivation.

## Introduction

Sleep occupies about a third of our lives, indicating its physiological importance (Somers, 2005; Walker, 2018). Although the restorative effect of sleep and the impairment of inadequate sleep are not fully understood, the fundamental role of sleep in maintaining physical, mental, and emotional health has been unanimously recognized. Moreover, the effects of sleep deprivation on cardiovascular, respiratory, and neurological activities have been extensively evaluated (Liew and Aung, 2021). However, physiological interaction changes in response to sleep deprivation remain obscure. Human physiological systems, which regulate their functions through interplays between one another are of great importance in maintaining physiological homeostasis. The physiological coupling among the cardiac, respiratory and cardiovascular systems under the central and autonomic regulation reflects the capacity of the body to adapt and function in an ever-changing environment (Garcia et al., 2013). Additionally, good physiological coupling has been associated with improvements in health, psychophysiological status, physical and cognitive performance as well as a general sense of well-being (Mejía-Mejía et al., 2018). Since the heartbeat, respiration, and blood pressure with common frequencies are under neuroautonomic control that regulates their complex dynamics and further influences their coupling through intrinsic feedback mechanisms at different time scales (Bartsch et al., 2010), identify and quantify the acute effects of sleep deprivation on physiological coupling is potentially helpful for elucidating the importance of sleep.

Sleep deprivation is associated with a range of negative physiological and psychological outcomes encompassing dysregulation of the autonomic nervous system (ANS), adverse cardiovascular events, and cognitive and complex motor performance impairment. In previous studies, it has been observed that acute total sleep deprivation (ATSD) may decrease parasympathetic cardiac modulation and/or increase sympathetic activity based on heart rate variability (HRV) analysis (Zhong et al., 2005; Chen et al., 2013; Glos et al., 2014; Virtanen et al., 2015; Westphal et al., 2021). However, other investigations had contradictory results that accumulation of acute sleep loss resulted in decreased heart rate and increased HRV, which reflects the enhancement of vagal outflow (Vaara et al., 2009; Skurvydas et al., 2021). Several studies also found that exposure to continuous ATSD appears to cause hemodynamic dysfunction through raising systolic blood pressure, diastolic blood pressure, arterial stiffness, or lowering baroreflex sensitivity (Robillard et al., 2011; Sunbul et al., 2014; Słomko et al., 2018). Different from the above research conclusions, one study showed that ATSD without significant additional stress or disturbances does not lead to increased arterial pressure values or changes in autonomic or baroreflex profiles (Pagani et al., 2009). Findings of a stress test simulating a military march also supported the contention that one-night sleep deprivation presents no obvious effects on physical and physiological responses (Nieto-Jiménez and Orellana, 2020). Moreover, deprivation of sleep has been known to affect brain functions (Patrick et al., 2017; Hudson et al., 2020; Garbarino et al., 2021; Grèzes et al., 2021). In addition, recent studies reported a decrease in respiratory motor output by altering its cortical component with a subsequent reduction of inspiratory endurance while being deprived of one-night sleep (Westphal et al., 2021). Most of these results suggest that ATSD may have an impact on the cardiovascular, brain, and respiratory systems. However, the studies supporting these findings are mostly conducted to evaluate the potential effects of sleep deprivation on a single physiological system, ignoring the coupling between complex systems.

As typical complex systems, physiological systems are composed of many interdependent agents regulated by intrinsic mechanisms in multiple spatial and temporal scales resulting in non-stationary and non-linear behavior. Analysis of intimately interrelated physiological systems provides information about the coupling from the dynamic, communicative network of interacting functions, leading to an improved understanding of the operation between systems under different physiological and pathophysiological conditions. The physiological coupling has been extensively assessed in previous studies to explore its physiological and pathophysiological relevance, and its relation to mechanisms of occurrence and progression of the disease (Bartsch et al., 2012; Mejía-Mejía et al., 2018; Naji et al., 2019). However, there are no studies on whether ATSD affects physiological coupling. By physiological coupling analysis of synchronous respiration, HRV, pulse transit time (PTT) and electroencephalography (EEG) data acquired from healthy young adults in resting state, the present study aimed to evaluate the potential effects of 24-h ATSD on cardiorespiratory, respiratory-cardiovascular, cardiovascular, and cortico-cardiac coupling. We hypothesized that 24-h ATSD in healthy young volunteers might alter physiological coupling, autonomic regulation and hemodynamics.

## Materials and methods

### Participants and study design

Healthy and physically active students from Beijing Sport University and Chinese PLA Medical School, performing moderate-intensity exercise 3–5 times per week, were included in the study between June 8, 2021, and September 10, 2021. All participants underwent a detailed medical history and physical examination. Cardiovascular status (diagnosed by GE Marquette Mac 5000 EKG system), hemodynamic characteristics (assessed by CNAP Monitor 500), the clinical hematological and biochemical tests (performed by Sysmex 9100 automated machine and Roche cobas 8000 system based on venous blood samples) were screened and checked. Exclusion criteria were the following: (1) with acute or chronic medical diseases such as hypertension, diabetes mellitus, sleep disorders, hyperlipidemia, etc; (2) use of medication known to influence sleep; (3) night work, shift work, or transmeridian travel 3 weeks before the study; (4) receiving any medications, and body mass index (BMI) ≥ 28.0 kg/m^2^; (5) daily alcohol and/or caffeine users, and smokers; (6) abnormal biological, physical and, physiological results based on examination; and (7) mean sleep duration in the week before the study less than 7 h.

Each participant underwent two sleep pressure periods: a night of regular sleep and a night of sleep deprivation. In the first period of undisturbed sleep night in their usual sleeping condition at home, all the subjects who participated in the experiment wore wrist actigraphy to record their sleep duration. During the following period of sleep deprivation, the participants of the experiment were admitted to the Department of Hyperbaric Oxygen of the Chinese PLA General Hospital at 8 am and stayed there until 11 am one day later. All subjects had a designated single ward with the temperature controlled at 22–25°C. In addition, no bed was provided during the night of sleep deprivation to prevent them from sleeping after lying down for a long time. The participants were instructed and guided to make sure that they understand the detailed experimental procedures. During the sleep deprivation period, all the subjects were allowed to engage in their daily routine preferred activities including watching videos, playing games, reading, writing, or doing their school work, but were prevented from vigorous physical activity, sleep, and intake of alcohol or stimulants such as caffeine. At all times of sleep deprivation, the sleep–wake states of the subjects were monitored by the shift duty nurses in the control room using a surveillance camera. Moreover, the shift nurses conducted a nighttime ward inspection every hour throughout the sleep deprivation period. When the subject exhibited signs of sleepiness, nurses kept the subject stay awake by calling his/her name, engaging in conversation, and playing games.

Continuous 10-min of resting-state physiological and hemodynamic data after regular sleep night and after 24-h ATSD were recorded in the morning hours (9:30–10:30 am) in a quiet chamber, respectively. During each data recording session, subjects were asked to close their eyes but stay awake and think of nothing in a supine position. To prevent the subjects from falling asleep, we chose a relatively short 10-min for data acquisition. The real-time EEG signal of the participants was monitored by the experimenter to ensure that they will not fall asleep.

The study was approved by the Institutional Review Committee of the Chinese PLA General Hospital, and all subjects gave informed consent in written form. The study was pre-registered at the Chinese Clinical Trial Registry (http://www.chictr.org.cn; ChiCTR2000033645; 06/07/2020).

### Data recording and preprocessing

Single-channel unipolar frontal pole electroencephalography (EEG), single-channel of standard leads II electrocardiogram (ECG), saturation of pulse oxygen (SpO_2_), photoplethysmography (PPG), and bio-electrical impedance signals were acquired simultaneously by a Biopac MP160 system (Biopac Sytem Inc Goleta, CA, United States). Electro-oculograms were also recorded from the left eye for EEG artifact removal. Physiological data were sampled at 2,000 Hz and exported in MAT format. Continuous hemodynamic variables include systolic blood pressure (SBP), diastolic blood pressure (DBP), mean arterial pressure (MAP), cardiac output (CO), cardiac index (CI), stroke volume variability (SVV), systemic vascular resistance (SVR), as well as arterial blood pressure (AP) were recorded non-invasively at a sampling frequency of 100 Hz using a CNAP Monitor 500 (CNSystems Medizintechnik, Graz, Austria). Hemodynamic data were output in TXT format. All acquired data were stored on a personal computer for further offline analysis.

**FIGURE 1:**
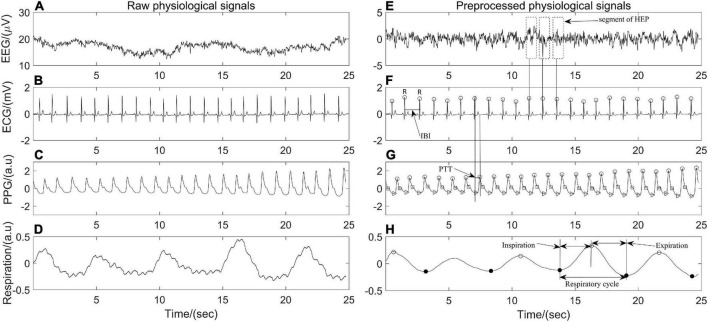
Twenty-five seconds of synchronous **(A–D)** raw electroencephalography (EEG), electrocardiography (ECG), photoplethysmography (PPG), respiration signals and **(E–H)** their corresponding preprocessed signals of one participant in the position of supine.

The physiological data were processed by Acqknowledge software version 5.0 (Biopac Sytem Inc., Goleta, CA, United States) and the hemodynamic variables were extracted by MATLAB R2020 (MathWorks, Natick, MA, United States). Raw EEG data were epoched into 2 s segments with no overlap and filtered with an 0.05–100 Hz bandpass filter. Then, a notch filter at 50 Hz was applied to remove power line interference, and artifact rejection was performed by visual inspection to remove the non-physiological artifacts. Independent component analysis was conducted to eliminate possible heartbeat artifacts, eye movement, eye blink, and muscle artifacts. All ECG and PPG recordings were analyzed with Kubios (Kubios 2.2, University of Eastern Finland, Kuopio), on which ECG R waves, PPG onsets and peaks were detected and labeled automatically. The interbeat interval (IBI) between 300 and 2000 ms, consecutive IBI differences ≤ 200 ms, and prolongations or shortenings ≤20% than the average of five preceding sinus rhythm IBIs were considered as sinus rhythm QRS complexes (Liu et al., 2020). Thereafter, automatic annotated results were carefully visually inspected and manually corrected by editing ectopic beats, arrhythmias and noise to suppress computational errors. Peaks of PPG signals were detected by matched filtering approach (Mejía-Mejía et al., 2018). In addition, each pair of PPG and ECG recordings acquired simultaneously was evaluated beat by beat. PTT, which is a measurement of the time it takes for an arterial pulse wave to reach the periphery and has been shown to approximate blood pressure (Hoshide et al., 2022), was estimated by detecting the time lapse between the R peak of ECG and the peak of PPG in the same heartbeat cycle. The respiratory signal was obtained by band-pass filtering thoracic bio-electrical impedance data with specified passband frequencies of 0.05 and 1 Hz (Figure 1). IBI, PTT time series and respiratory signals were resampled at 5 Hz for further analysis.

### Physiological coupling analysis

#### Magnitude squared coherence

**FIGURE 2:**
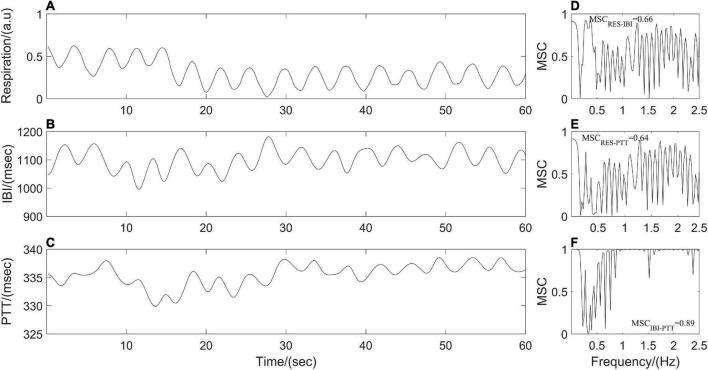
**(A–C)** Resampled respiration, interbeat interval (IBI), and pulse transit time (PTT) time series from one participant. Panels **(D–F)** represent cardiorespiratory, respiratory-cardiovascular, and cardiovascular magnitude squared coherence (MSC), respectively.

The magnitude squared coherence (MSC) analysis is a linear dependency signal processing technique that returns real values between 0 and 1 to determine if two signals *x*(*t*) and *y*(*t*) have similar oscillatory activity with each other, and is often used to quantify coupling strength between oscillatory physiological signals (Stoica and Moses, 1997; McCraty, 2017; Malekpour et al., 2018). The MSC of signals *x*(*t*) and *y*(*t*) at a given frequency *f* is obtained by the Equation 1


(1) $$M\,S\,C\,\left(f\right)=\frac{{\left|S_{x\,y}\,\left(f\right)\right|}^{2}}{S_{x\,x}\,\left(f\right)•S_{y\,y}\,\left(f\right)}$$

where *S*_*xy*_ is the cross-power spectral density of *x*(*t*) and *y*(*t*) at frequency *f*. *S*_*xx*_ and *S*_*yy*_ are the auto-power spectral densities of *x*(*t*) and *y*(*t*), respectively. *MSC(f)* = 1 for all frequencies *f* if and only if *x*(*t*) and *y*(*t)* are related through a linear time-invariant system, that is, the two signals are linearly dependent or completely coupled over time. While *MSC(f)* = 0 means that *x*(*t*) and *y*(*t*) are not linearly related or decoupled over time (Stoica and Moses, 1997; Malekpour et al., 2018). The synchronous respiratory, IBI and PTT signals in the present study were divided into segments with the same length (1 min), the power spectral density and the cross-power spectral density are estimated by Welch’s average method between each pair of segments (respiration and IBI; respiration and PTT; IBI and PTT) before averaging them to obtain the *MSC(f)* values at different frequencies. Finally, the mean (*MSC*_*RES–IBI*_, *MSC*_*RES–PTT*_, and *MSC*_*IBI–PTT*_) over the *MSC(f)* values for all frequencies of the frequency band were quantified as the coupling strength among respiratory and cardiovascular systems (Figure 2).

#### Phase synchronization index

**FIGURE 3:**
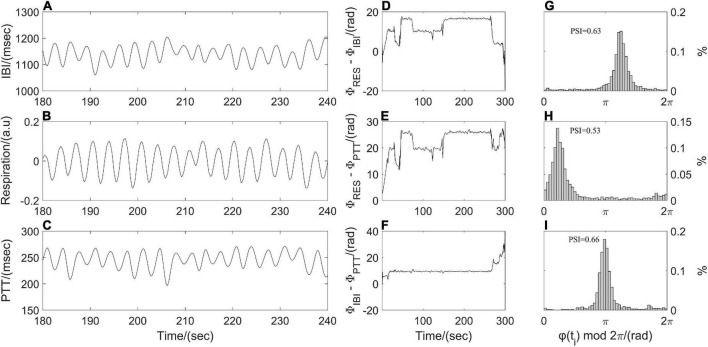
Illustration of phase synchronization analysis. **(A)** Interbeat interval (IBI) time series, **(B)** respiratory signal, **(C)** pulse transit time (PTT) time series; instantaneous phase differences **(D)** between respiratory signal and IBI time series, **(E)** between respiratory signal and PTT time series, **(F)** between IBI and PTT time series; and the distribution of *φ(t_*i*_)* mod 2π **(G)** between respiration and IBI, **(H)** between respiration and PTT, **(I)** between IBI and PTT.


The phase synchronization index (PSI) was often used to quantify the coupling strength between respiratory and cardiovascular systems (Zheng et al., 2016; Mejía-Mejía et al., 2018). Resampled respiratory, IBI, and PTT time series were processed by empirical mode decomposition to extract the dominant component and then were segmented into subsections with the same length (1 min). Following by Hilbert transform, the instantaneous phases of the dominant component of synchronous IBI, PTT and respiratory time series, that is, Φ*_IBI_ (t_i_)*, Φ*_PTT_ (t_i_)*, and Φ*_RES_ (t_i_)* were computed. As shown in Figure 3, a point-by-point phase difference *φ(t_i_)* between two instantaneous phases was calculated (IBI and PTT: *φ(t_i_)* = Φ*_IBI_ (t_i_)* - Φ*_PTT_ (t_i_)*; IBI and respiration: *φ(t_i_)* = Φ*_RES_ (t_i_)* - Φ*_IBI_ (t_i_)*; PTT and respiration: *φ(t_i_)* = Φ*_RES_ (t_i_)* - Φ*_PTT_ (t_i_)*). Phase synchronization between each pair of segments was quantified by the Equation 2 before averaging them to obtain PSI.

(2) $$P\,S={<\cos\left(\varphi \,\left(t_{i}\right)\right)>}^{2}+{<\sin\left(\varphi \,\left(t_{i}\right)\right)>}^{2}$$

where brackets denote an average. The theoretical value for PSI is ranging from 0 to 1. A larger value of PSI indicates higher synchronization between two time series, and with a narrower range of phase difference distribution (Figure 3). For two time series in the real world, a constant phase difference, that is, PSI = 1 indicates a complete synchronization. while PSI < 0.14 may be considered completely desynchronized (Cysarz and Büssing, 2005).

#### Heartbeat evoked potential amplitudes

**FIGURE 4:**
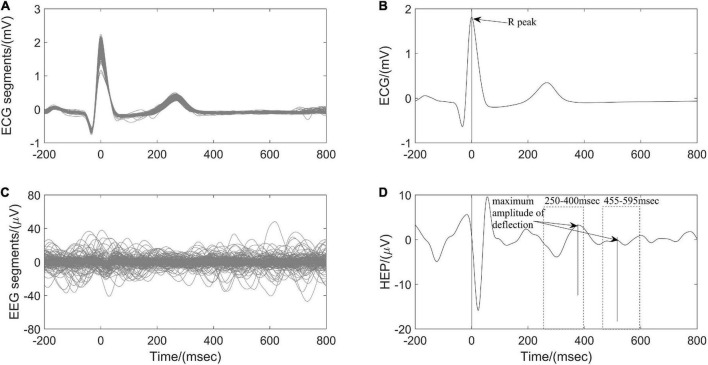
Computation of heartbeat evoked potential (HEP) for one participant According to **(A)** the detected R peaks of electrocardiography (ECG) signals, **(B)** corresponding electroencephalography (EEG) brain activity is aligned. **(C)** The HEP is obtained by averaging over all clean EEG signals time-locked to **(D)** ECG R peaks.

Heartbeat evoked potential (HEP) can be used to identify the specific mechanism of afferent input from the heart to the brain during different physiological states and may serve as an indication of brain-heart coupling (Park and Blanke, 2019). After preprocessing ECG and EEG as described above (Figure 1), resulting continuous EEG data were segmented relatively to the detected R peaks of ECG signal in epochs ranging from 200 ms before the R peaks to 800 ms after the R peaks. Segments of R-peak-triggered EEG were aligned and averaged for the computation of HEP. All segments were manually reviewed for artifacts identification and sweeps with EEG activity above 50 μV were excluded from further analysis. In addition, a baseline correction was also performed by subtracting the mean of the first 75 ms before the R peak from the entire HEP average for each individual (Montoya et al., 1993). Finally, the maximum amplitude of positive deflection and mean amplitude of each average HEP curve within the time interval 250–400 ms and 455–595 ms, respectively, after the R peak were determined (Figure 4).

#### HRV analysis

Traditional techniques of HRV analysis are grouped into the time domain, frequency domain, and non-linear methods. The time-domain measures including mean interbeat intervals (Mean IBI), the standard deviation of the heartbeat intervals (SDNN), and the square root of the mean of the sum of squares of the differences between adjacent heartbeat intervals (RMSSD) were calculated to represent the total variance and vagal modulation of heart rate (Task Force., 1996). Based on the Fast Fourier transform spectrum, the frequency domain measures were computed from the power spectral density estimate for each frequency band including absolute power values of low frequency (LF, 0.04–0.15 Hz), high frequency (HF, 0.15–0.40 Hz) and LF/HF power ratio (Task Force., 1996). The LF and HF were also transformed in natural logarithmic (ln) value. Non-linear measures of approximate entropy (ApEn) and sample entropy (SampEn) were also taken into consideration to characterize the single-scale complexity or regularity of the HRV time series by measuring the unpredictability of fluctuation patterns (Richman and Moorman, 2000).

#### EEG analysis

The resulting EEG signals were filtered into four different frequency bands: δ 0.5–4 Hz, θ 4–7 Hz, α 8–13 Hz, and β 14–30 Hz. Welch’s periodogram method was used to estimate the power spectral density with non-overlapping Hanning windows of 4 s. The power spectral density of the δ, θ, α, and β bands were calculated by an average computation. EEG band power ratios α/β and (θ + α)/β were also computed to quantify fatigue (Dissanayake et al., 2022).

### Statistical analysis

All data are presented as Mean ± standard deviation for continuous variables. Statistical analyses were performed using the SPSS version 20 software package (SPSS, Chicago, IL, United States). Gaussian distribution and homogeneity of variance tests were applied to determine the distribution and homoscedasticity of sample data. As a result of the non-normal distribution and heterogeneity of variance of some sample data, a Wilcoxon signed-rank test was applied to compare the differences in physiological coupling indices, EEG features, HRV measurements and hemodynamic variables between baseline states and after 24-h ATSD. Multiple linear regression analyses were also employed to identify potential co-factors related to physiological coupling indices changes after 24-h ATSD. The goodness of fit of the test and reference regression line was quantified by the coefficient of determination R^2^. All the p values were adjusted using the false discovery rate (FDR) method and a value of *p* < 0.05 was considered to indicate statistical significance.

## Results

Five subjects were excluded from the final analysis, of which three subjects had incomplete physiological signals and the other two subjects had abnormal physical examination results. Finally, a total of 38 (23 males and 15 females) healthy volunteers, aged 23.2 ± 2.4 years and with a BMI of 22.03 ± 2.55 kg/m^2^, were enrolled in the present study. All participants reported a normal sleep pattern over the previous week. The average nightly amount of regular sleep before the 24-h ATSD was 7.7 ± 0.4 h, and the mean duration of sleep deprivation was 24.2 ± 1.8 h. Cardiovascular status, hemodynamic characteristics, and hematological as well as biochemical parameters of all the 38 subjects showed ostensibly normal. Demographics and basic clinical characteristics of the study population are presented in Table 1.

**TABLE 1:** Demographics and basic clinical characteristics of the study population.

Variables	*N* = 38
Age (years)	23.2 ± 2.4
Gender (%)	Male (60.5%)
	Female (39.5%)
BMI (kg/m^2^)	22.03 ± 2.55
SBP (mmHg)	108 ± 13
DBP (mmHg)	66 ± 11
MAP (mmHg)	82 ± 10
Heart rate (bpm)	66 ± 11
SpO_2_ (%)	96 ± 1
Hemoglobin (g/L)	141 ± 13
Red blood cell (×10^12^/L)	4.69 ± 0.47
White blood cell (×10^9^/L)	6.37 ± 1.42
Platelets (×10^9^/L)	232 ± 58
Glucose (mmol/L)	5.3 ± 0.8
Uric acid (μmol/L)	341.2 ± 82.2
Cholesterol (μmol/L)	4.00 ± 0.70
Creatine kinase (mmol/L)	132.4 ± 100.3
Potassium (mmol/L)	4.12 ± 0.24
Cystatin C (mg/L)	0.74 ± 0.11
Regular sleep (h)	7.7 ± 0.4
Sleep deprivation (h)	24.2 ± 1.8

BMI, body mass index; SBP, systolic blood pressure; DBP, diastolic blood pressure; MAP, mean arterial pressure; SpO_2_, saturation of pulse oxygen.

**FIGURE 5:**
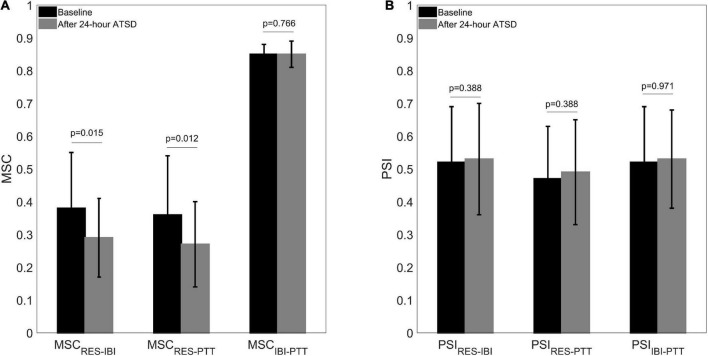
**(A)** Cardiorespiratory, respiratory-cardiovascular, and cardiovascular magnitude squared coherence (MSC) as well as **(B)** phase synchronization index (PSI) before and after 24-h acute total sleep deprivation (ATSD) for healthy volunteers.

**FIGURE 6:**
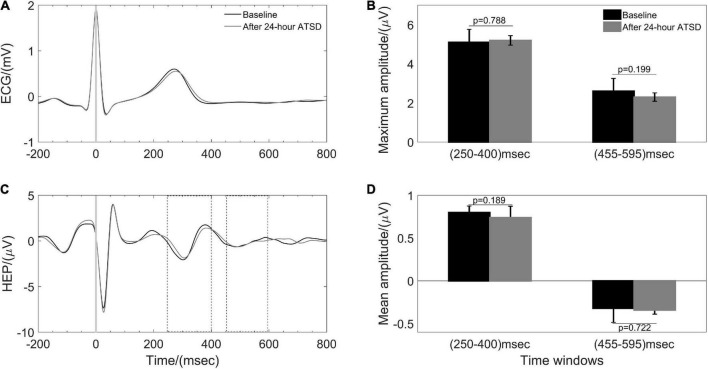
Heartbeat evoked potential (HEP) during baseline and after 24-h acute total sleep deprivation (ATSD). **(A)** The averaged segments of heartbeats and **(B)** the HEP waveforms in response to the R peak averaged for 38 participants. Bar graphs showing the **(C)** maximum and **(D)** mean amplitude for 250–400 ms and 455–595 ms time windows of HEP for baseline and 24-h ATSD period, respectively.

Figures 5, 6 illustrate physiological coupling indices (MSC, PSI, and HEP amplitudes) for all the participants at both baseline and 24-h ATSD. Compared with baseline, the MSC_RES–IBI_ (0.38 ± 0.17 vs. 0.29 ± 0.12, *p* = 0.015) and MSC_RES–PTT_ (0.36 ± 0.18 vs. 0.27 ± 0.13, *p* = 0.012) were significantly decreased after 24-h ATSD (Figure 5). No significant changes were observed for MSC_IBI–PTT_ (0.85 ± 0.03 vs. 0.85 ± 0.04, *p* = 0.766), PSI_RES–IBI_ (0.52 ± 0.17 vs. 0.53 ± 0.17, *p* = 0.388), PSI_RES–PTT_ (0.47 ± 0.16 vs. 0.49 ± 0.16, p = 0.388), and PSI_IBI–PTT_ (0.52 ± 0.17 vs. 0.53 ± 0.15, *p* = 0.971) after 24-h ATSD compared with baseline. As shown in Figure 6, the maximum amplitude of positive deflection for 250–400 ms (5.1 ± 4.1 μV vs. 5.2 ± 4.0 μV, *p* = 0.788) and 455–595 ms (2.6 ± 1.5 μV vs. 2.3 ± 1.3 μV, *p* = 0.199) time windows of HEP showed no significant differences before and after sleep deprivation. Similarly, the mean amplitudes for the aforementioned 250–400 ms (0.8 ± 0.1 μV vs. 0.7 ± 0.1 μV, *p* = 0.189) and 455–595 ms (–0.3 ± 0.1 μV vs. –0.3 ± 0.1 μV, *p* = 0.722) time windows of HEP also had no significant statistical differences.

As shown in Tables 2, 24-h ATSD did not change the time domain (Mean IBI, SDNN, and RMSSD), frequency domain (LF, HF, logLF, logHF, and LF/HF) and non-linear (ApEn and SampEn) HRV parameters (all *p* > 0.05). The power spectral density of δ, θ, α, and β power bands of EEG after 24-h sleep deprivation showed no significant differences from those after normal sleep (all *p* > 0.05). In addition, sleep deprivation did not significantly affect the EEG power band ratios of α/β and (θ + α)/β (all *p* > 0.05). In addition, no obvious alterations were observed in hemodynamic measurements (SYS, DIA, MAP, CO, CI SVV, and SVR) for the study population in the supine position related to the 24-h ATSD (Table 3).

**TABLE 2:** Mean ± standard deviation of heart rate variability (HRV) parameters and electroencephalography (EEG) band power ratios in the supine position before and after 24-h acute total sleep deprivation (ATSD) for 38 healthy participants.

Variables	Baseline (*N* = 38)	After 24-h ATSD (*N* = 38)	*p*
Mean IBI (ms)	955 ± 136	994 ± 166	0.070
SDNN (ms)	59 ± 18	68 ± 24	0.050
RMSSD (ms)	52 ± 22	60 ± 31	0.070
LF (ms^2^)	838 ± 688	1120 ± 904	0.088
HF (ms^2^)	1058 ± 837	1416 ± 1360	0.060
log LF	6.35 ± 0.83	6.58 ± 0.91	0.139
log HF	6.59 ± 0.96	6.82 ± 0.98	0.070
LF/HF	1.17 ± 1.35	1.14 ± 1.04	0.890
ApEn	0.94 ± 0.09	0.93 ± 0.10	0.505
SampEn	1.91 ± 0.26	1.88 ± 0.29	0.791
δ power (log(μV^2^/Hz))	2.32 ± 2.21	2.29 ± 3.30	0.323
θ power (log(μV^2^/Hz))	0.16 ± 0.16	0.12 ± 0.17	0.057
α power (log(μV^2^/Hz))	0.03 ± 0.02	0.03 ± 0.02	0.394
B power (log(μV^2^/Hz))	0.02 ± 0.02	0.01 ± 0.01	0.057
α/β	1.91 ± 1.42	2.29 ± 1.37	0.134
(θ + α)/β	11.23 ± 11.98	11.47 ± 9.62	0.856

IBI, interbeat intervals; SDNN, standard deviation of the heartbeat intervals; RMSSD, square root of the mean of the sum of squares of the differences between adjacent heartbeat intervals; LF, low frequency (0.04–0.15 Hz); HF, high frequency (0.15–0.40 Hz); ApEn, approximate entropy; SampEn, sample entropy; θ, (4–7) Hz band of EEG; α, (8–13) Hz band of EEG; β, (14–30) Hz band of EEG.

**TABLE 3:** Mean ± standard deviation of hemodynamic measurements in the supine position before and after 24-h acute total sleep deprivation (ATSD) for healthy volunteers.

Variables	Baseline (*N* = 38)	After 24-h ATSD (*N* = 38)	*p*
SBP (mmHg)	107 ± 14	106 ± 17	0.361
DBP (mmHg)	62 ± 10	62 ± 13	0.744
MAP (mmHg)	79 ± 10	79 ± 14	0.896
CO (L/min)	6.6 ± 1.6	6.3 ± 1.0	0.372
CI (L/min m^2^)	3.8 ± 0.9	3.6 ± 0.8	0.313
SVV (%)	12.61 ± 2.92	11.83 ± 4.00	0.243
SVR (dyn s/cm^5^)	1023 ± 282	1065 ± 292	0.313

SBP, systolic blood pressure; DBP, diastolic blood pressure; MAP, mean arterial pressure; CO, cardiac output; CI, cardiac index; SVV, stroke volume variability; SVR, systemic vascular resistance.

In multivariate linear regression analyses, significanltly decreased physiological coupling indices MSC_RES–IBI_, MSC_RES–PTT_ and their variations ΔMSC_RES–IBI_ and ΔMSC_RES–PTT_ did not signifificantly correlate with clinical/demographic data such as gender, age, BMI, sleep duration, hemodynamic characteristics, and hematological as well as biochemical parameters (all *p* values for β coefficients > 0.05 shown in Table 4). All the analyzed baseline factors were not independent factors associated with MSC_RES–IBI_, MSC_RES–PTT_ and their variations in the final models (all *p* > 0.05).

**TABLE 4:** Multiple linear regression models predicting significantly changed physiological coupling indices.

Predictors	Significantly changed physiological coupling indices					
	MSC_RES–IBI_ (Baseline)	MSC_RES–IBI_ (ATSD)	ΔMSC_RES–IBI_	MSC_RES–PTT_ (Baseline)	MSC_RES–PTT_ (ATSD)	ΔMSC_RES–PTT_
	R^2^ = 0.595	R^2^ = 0.429	R^2^ = 0.416	R^2^ = 0.583	R^2^ = 0.421	R^2^ = 0.412
Gender	0.068	0.115	0.047	0.07	0.121	0.05
Age (years)	–0.004	–0.009	–0.005	–0.003	–0.007	–0.004
SBP (mmHg)	–0.007	–0.002	0.005	–0.007	–0.001	0.006
DBP (mmHg)	–0.002	1.58E-05	0.002	–0.001	0	0.001
MAP (mmHg)	0.009	–3.04E-05	–0.009	0.008	–0.001	–0.009
Heart rate (bpm)	0.002	–0.001	–0.003	0.001	–0.001	–0.002
SpO_2_ (%)	0.013	0	–0.012	0.01	0.001	–0.008
Regular sleep (hour)	0.156	–0.081	–0.237	0.158	–0.087	–0.246
BMI (kg/m^2^)	0.015	–0.002	–0.017	0.015	–0.004	–0.019
Hemoglobin (g/L)	–0.011	–0.004	0.007	–0.01	–0.005	0.005
Red blood cell (*10^12^/L)	0.167	0.042	–0.125	0.131	0.051	–0.079
White blood cell (*10^9^/L)	–0.003	0.006	0.009	–0.01	0.007	0.017
Platelets (*10^9^/L)	–0.001	0	0.001	–0.001	0	0.001
Glucose (mmol/L)	–0.071	–0.057	0.015	–0.072	–0.07	0.002
Uric acid (μmol/L)	–6.68E-05	0	0	–6.11E-06	0	0
Cholesterol (μmol/L)	0.055	0.062	0.007	0.064	0.065	0.002
Creatine kinase (mmol/L)	0	5.01E-06	–9.91E-05	0	6.29E-06	0
Potassium (mmol/L)	0.281	0.035	–0.246	0.266	0.038	–0.228
Cystatin C (mg/L)	–0.127	0.024	0.15	–0.13	0.018	0.15

BMI, body mass index; SBP, systolic blood pressure; DBP, diastolic blood pressure; MAP, mean arterial pressure; SpO_2_, saturation of pulse oxygen. R^2^ are squared multiple correlation of the entire regression model. The values are unstandardized regression coefficients β of the predictors with *p < 0.05, **p < 0.01, and ***p < 0.001.

## Discussion

The present study demonstrates that 24-h ATSD affects the coupling between physiological systems. The main findings suggest that 24-h ATSD significantly decreased the strength of cardiorespiratory and respiratory-cardiovascular coupling while having no obvious effects on cardiovascular and cortico-cardiac coupling as well as the activity of ANS. To the best of our knowledge, this is the first study to reveal the effects of 24-h ATSD on physiological coupling based on MSC, PSI, and HEP analysis.

Complex living systems, especially human beings, are composed of numerous interacted, dynamic networks of biological structures, subsystems and processes (McCraty and Childre, 2010). The maintenance of health is inseparable from homeostasis in the human body and the multi-scale coupling, which regulates synchronized activity in the networks among the structures and subsystems (McCraty and Zayas, 2014). It has long been recognized that there is an important relationship between sleep and human physical and mental health (Somers, 2005; Walker, 2018). Sleep is one of the most basic biological activities which is crucial for both high-level cognitive processing and also basic maintenance and restoration of physiological function (Simpson and Dinges, 2007; Fultz et al., 2019), and while in sleep the brain, heart rate and breathing will exhibit sleep-related characteristics (Purves et al., 2011). Previous studies suggested that sleep deprivation may affect the function of the central nervous system, ANS, cardiovascular system, and respiratory system (Zhong et al., 2005; Vaara et al., 2009; Robillard et al., 2011; Chen et al., 2013; Glos et al., 2014; Sunbul et al., 2014; Virtanen et al., 2015; Słomko et al., 2018; Skurvydas et al., 2021; Westphal et al., 2021).

Physiological coupling is reflected by ordered patterns related to increased vagally mediated HRV, entrainment among heartbeat, blood pressure and respiration, and improved EEG-ECG synchronization (McCraty and Zayas, 2014). It is also well known that the coupling or synchronization between heartbeat and blood pressure is also regulated by respiration through neural effects on cardiac vagal activity and mechanical effects on stroke volume and intrathoracic pressure (Mejía-Mejía et al., 2018). Desynchronizations between cardiorespiratory coupling and/or cardiovascular coupling were reported following hypertension, myocardial infarctions, depression, obstructive sleep apnea and acute insomnia (Shigdel et al., 2019; Zhao et al., 2019; Delesie et al., 2021). In the present study, we found declined coupling strength of cardiorespiratory and respiratory-cardiovascular of healthy young subjects after 24-h ATSD. Our findings are not only almost in agreement with previously reported results of disease and adverse interventions weakening physiological coupling strength, but also provide evidence to support our hypothesis. The cardiovascular control system is a closed-loop control system, where the effect of the heartbeat on the blood pressure is mainly mechanical whereas the reversal effect is mostly mediated by ANS *via* the baroreceptor reflex (Pereda et al., 2005). The cardiovascular coupling strength quantified by MSC_IBI–PTT_ and PSI_IBI–PTT_ in our study was kept unchanged, indicating a stable state of autonomic regulation. The result is supported by our findings on HRV and hemodynamic parameters, which are used to assess the functional state of ANS. The negative results may also be confounded by the status of the subjects during data acquisition, especially after 24-h ATSD. Although we have taken measures, it is difficult for the participants to keep constant awake. As uncovered by previous studies (Bartsch et al., 2010, 2012), healthy subjects show a change in the degree of cardio-respiratory phase synchronization during different sleep stages, with a relatively low phase synchronization during rapid eye movement sleep and wake state, higher synchronization during light sleep and highest during deep sleep. These results show a remarkable sensitivity of the cardiorespiratory coupling in response to sleep-stage transitions. Although the characteristic parameters of the δ, θ, α, and β frequency bands of EEG have not changed significantly before and after 24-h ATSD, we can not completely exclude the interference of the subjects’ state during the data acquisition process, which is also the aspect where the further study needs to be improved.

Cortico-cardiac synchronization quantified by HEP characteristics is considered to be a marker of interoception reflecting the cortical processing of afferent cardiac signals (Park and Blanke, 2019). Abundant evidence indicates that the cortico-cardiac coupling is altered in different physiological or pathological conditions of emotional dysregulation including anxiety disorder, borderline personality disorder and post-traumatic stress disorder (Pang et al., 2019; Flasbeck et al., 2020; Schmitz et al., 2021; Bogdány et al., 2022). The negative results of our study may be due to that the recruited subjects are highly qualified physically active students, who have increased their resilience and ability to better self-regulate stress and reached a more coherent state with reinforced performance. It is postulated that the stressor or challenge of 24-h ATSD may not be intense enough to perturb the stability of cortico-cardiac and cardiovascular coupling or produce a mismatch between the input pattern of new experience and previously established neural programs to induce a significant change of activity in the central nervous system and ANS. The observed invariable fatigue quantified by α/β and (θ + α)/β from EEG before and after 24-h ATSD, provides indirect evidence for the above inference.

Furthermore, the effects of ATSD on cardiovascular autonomic modulation have been comprehensively studied and reported discrepant results. In the present study, all the analyzed HRV parameters and hemodynamic measurements were kept unchanged during 24-h ATSD, indicating the highly stable state of hemodynamic and cardiac autonomic modulation. These findings are in agreement with previous reports of a comparable cardiovascular autonomic after acute sleep deprivation (Zhong et al., 2005; Pagani et al., 2009; Nieto-Jiménez and Orellana, 2020). However, sleep deprivation is often considered a stressor and is believed to deteriorate autonomic and cardiovascular function. In most of the relevant previous studies, sleep deprivation is associated with a decrease in parasympathetic/vagal modulation and baroreflex sensitivity, and an increase in sympathetic modulation and arterial pressure or its variability (Zhong et al., 2005; Chen et al., 2013; Glos et al., 2014; Virtanen et al., 2015; Westphal et al., 2021). In contrast, very few studies have found that sleep deprivation influences cardiovascular autonomic response by increasing parasympathetic modulation (Vaara et al., 2009; Skurvydas et al., 2021). Taken together these observations suggest that previous findings of changed HRV and blood pressure or its variability after 24-hour ATSD might rather reflect the disturbing effect of psychological stress on autonomic function (Pagani et al., 2009). In the present study, the cardiovascular autonomic function of participants was evaluated in the supine position, which may attenuate the force of gravity on blood volume redistribution, affect respiratory resistance and cardiac output, and subsequently weaken the compensatory reflexive responses compared to the upright position (Cheshire and Goldstein, 2019). Besides posture, these inconsistent and even contradictory conclusions are likely due to the heterogeneous participants, sleep deprivation pattern, data length for analysis, physical activity, baseline sleep characteristics, circadian rhythmicity, and other factors that affect indices of the physiological signal, which make their interpretation difficult.

Several limitations need to be considered within this study. First, no control group was recruited to eliminate the possible confounding factors to guarantee that 24-h ATSD was primarily responsible for the observed findings. The study would have benefited from a counterbalanced order of the nights of sleep deprivation and undisturbed sleep, with a one-week washout period between the two measurements. Such a within-subject design would be preferable to include a control group as described in a previous study investigating cortical excitability/net synaptic strength and long-term potentiation-like plasticity (Kuhn et al., 2016). Second, the physiological signal of participants for baseline and 24-h ATSD sessions was recorded under spontaneous breathing conditions. Although there was no significant change in the respiratory rate before and after sleep deprivation, breathing patterns with inter-and/or intra-individual variability would probably mask the effects of sleep deprivation on physiological coupling and cardiovascular autonomic function. Third, the lack of objective sleep measures for baseline sleep quality assessment, the single-channel frontal EEG recording, and the change of living environment from spending the night at home versus at the hospital may affect the reliability of the present findings. Fourth, the recruited subjects are highly qualified physically active students with a small sample size, and this would be an obstacle to the generalization of our results.

## Conclusion

In summary, the 24-h ATSD causes perturbations in physiological interactions represented by decreased cardiorespiratory and respiratory-cardiovascular coupling strength, although cardiovascular coupling, cortico-cardiac synchronization and autonomic regulation were maintained. These findings suggest that physiological coupling analysis may serve as a sensitive and complementary approach for providing a thorough new insight into the complex effects of sleep deprivation. Further studies performed with standardized protocols on a larger scale in risky cohorts are warranted to clarify the potential effects of acute sleep deprivation on physiological coupling.

## Data availability statement

The raw data supporting the conclusions of this article will be made available by the authors, without undue reservation.

## Ethics statement

The studies involving human participants were reviewed and approved by Institutional Review Committee of Chinese PLA General Hospital. The patients/participants provided their written informed consent to participate in this study.

## Author contributions

HL proposed the scientific problem, designed the experiments, and wrote the draft manuscript. XY, GW, and YH collected the experimental data. HL and GW processed the physiological data and conducted the statistical analysis. HL and WW supervised the work and contributed to the revision and final version of the manuscript. All authors contributed to the article and approved the submitted version.
